# What’s in a Name? Some Early and Current Issues in Dendritic Cell Nomenclature

**DOI:** 10.3389/fimmu.2015.00267

**Published:** 2015-05-29

**Authors:** David Vremec, Ken Shortman

**Affiliations:** ^1^The Walter and Eliza Hall Institute, Melbourne, VIC, Australia; ^2^Department of Medical Biology, The University of Melbourne, Melbourne, VIC, Australia; ^3^Burnet Institute, Melbourne, VIC, Australia

**Keywords:** dendritic cells, DC subsets, monocytes, macrophages, nomenclature

The name dendritic cell (DC) was given by Steinman to describe the unusual cell type he saw in spleen cell suspensions. This morphological description is not sufficient to specify the cell of so much interest to immunologists; many cells can adopt a similar form. A useful functional definition evolved as Steinman and colleagues explored the immunological properties of this novel cell type (1). DCs were considered as antigen collecting and processing cells able to present antigen on MHC molecules and efficiently activate even primary T-cells. Nowadays, immunologists would likely add to this definition, a capacity to sense the context in which the antigen was collected, via receptors for pathogen or damaged cell-derived material. Why might we need to go beyond the name “dendritic cell” for cells with these well-understood functions? Some limitations of this single name arose early in DC research. This article surveys some problems of definition encountered in past work from our own laboratory. The problems we encountered arose from two sources, the first the discovery of different DC subsets and the need to determine whether these represented different maturation states or separate sub-lineages. The second was the difficulty in distinguishing these DC subsets from macrophages.

Our first hint that there could be distinct types of DCs came from our studies with Wu and Ardavin on thymic T and DC development (2). We were surprised to find that a high proportion of mouse thymic DCs stained with antibodies against characteristic T-cell markers, such as CD8α; it was a relief to find they did not stain with antibodies against CD3 or the T-cell receptor! Pickup of material from thymocytes was eliminated as an explanation. We then found a similar but less frequent DC subset staining for surface CD8α among the DCs in mouse spleen and these DCs were shown to express mRNA for CD8α (3). Others had already reported some staining of DCs with anti-CD8; our work emphasized that these CD8^+^ DCs were a distinct population, CD8α expression being positively correlated with expression of DEC205 but inversely correlated with expression of other markers such as CD4, CD11b, and, as illustrated in Figure 1, SIRPα (4, 5).

**Figure 1:**
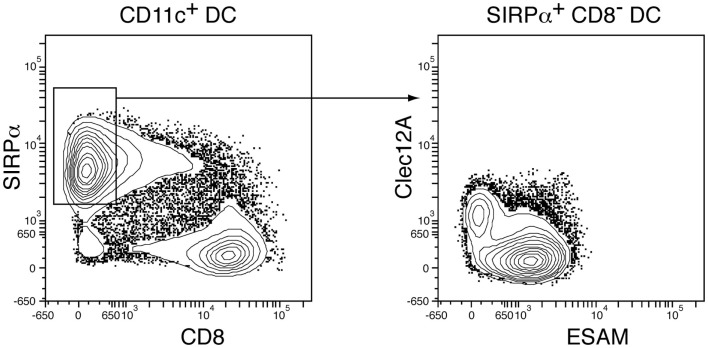
**Segregation of conventional DC subsets in mouse spleen**. Spleen CD11c^+^cDC were isolated, enriched, and gated as in Vremec et al. (5). Staining for CD8α and SIRPα allows clear segregation into the DC1 (CD8^+^ DC) and DC2 (CD8^−^ DC) subsets. However, the DC2 population can be separated into two further subsets by several surface markers, here Clec12A and ESAM as demonstrated in Lewis et al. (6).

Immunological interest in the CD8^+^ and CD8^−^ DC subsets increased when it became apparent from the work of many laboratories that these DCs differed in immunological functions. Differences were apparent in the expression of toll-like and other microbial pattern recognition receptors, in the cytokines produced on activation, in the fate of the T-cells they stimulated, in their capacity to phagocytize dead cells, and in the processing of antigens for MHC class I versus MHC class II presentation [reviewed in Ref. (7)]. The key findings from our laboratory were that the CD8^+^ DCs, when appropriately stimulated, were the most potent producers of IL12p70 (8), and that the CD8^+^ DCs have a strong bias to cross-presenting exogenous antigens, both soluble and particulate, for MHC class I presentation (9, 10).

An important issue became whether these functionally distinct DC types represented different lineages, or were simply different maturation states within one very plastic lineage. There was direct evidence, confirmed by us, that some CD8^−^ DCs could on adoptive transfer, produce CD8^+^ DCs. However, these CD8^+^ DCs proved to be generated from a small number of early members of the CD8^+^ DC lineage that had not yet acquired CD8α expression; the bulk of CD8^−^ DCs did not give rise to CD8^+^ DCs (11). Although sequential maturation states were found within the CD8^+^ DC lineage, with early forms lacking CD8α expression (12) and the earliest stages lacking capacity for antigen cross-presentation (13), there was a clear developmental separation from the bulk of CD8^−^ DCs. A further distinct DC type in mouse spleen became evident when the mouse equivalent of the human type 1 interferon-producing plasmacytoid dendritic cell (pDC) was identified (14). Although some pDCs expressed CD8α (15), they were clearly a separate lineage from the CD8^+^ conventional DCs (cDCs). Our subsequent work with Naik showed that spleen CD8^+^ DCs and CD8^−^ DCs represented separate cDC sub-lineages derived via pre-DC populations from a bone marrow pro-DC or common dendritic cell precursor (CDP) (16–18). Thus, because of differences in surface phenotype, immunological functions, and developmental pathways, these two spleen cDC populations had to be distinguished, and the terms CD8^+^ cDC and CD8^−^ cDC became current.

In line with the pioneering work of Salomon et al. (19) and Anjuere et al. (20), we extended our analysis of DC subtypes from mouse spleen to mouse lymph nodes (LNs) (4, 21). Spleen should contain only what we termed the “lymphoid tissue resident” cDCs whereas LNs should contain both these and the “migratory” cDC type arriving via the lymph from other tissues. It was immediately apparent that the level of DC heterogeneity was greater than in spleen. One source of heterogeneity was the existence of different activation states within even one DC lineage. In particular, the DC that had migrated even in steady state from peripheral tissues such as skin into LNs were more activated than those remaining in skin, and more activated than their lymphoid tissue resident counterparts; the DCs that had migrated expressed higher surface levels of MHC class II and of co-stimulator molecules such as CD86. Although they were first called “mature” DCs they proved to be not fully activated but “semi-mature”; they were not producing cytokines and were likely tolerogenic (22). A similar transformation termed “spontaneous activation” occurred when spleen cDCs were isolated and placed in culture (23). In both cases, further signals, such as given by microbial products interacting with TLR ligands, were required to produce a fully active, cytokine secreting immunogenic DC. However, even when these different activation states were considered, further cDC subsets not found in spleen were apparent, such as the epidermal Langerhans cell-derived LN DCs. The full lineage complexity of LN has now been well delineated by other laboratories, a notable finding being the existence of a migratory form of the CD8^+^ DC lineage but lacking CD8α expression, commonly termed as the CD103^+^ cDCs (24–26).

Our second problem with DC nomenclature arose as we attempted to distinguish DCs from macrophages, a particularly difficult exercise in inflamed tissues. It was also difficult to relate the DC populations we isolated from steady state mouse spleen with the DCs produced by culture of monocytes with granulocyte-macrophage colony stimulating factor (GM-CSF), a well-established model of DC generation (27). At that time, it was generally assumed that all DCs and macrophages would be bone marrow derived cells, with monocytes as the common late precursor. Some questioned whether it was valid even to consider DCs as a separate entity rather than as a macrophage variant (28). We had some sympathy with this view, since in experiments with Metcalf we had difficulty in segregating DCs from macrophages in the peritoneal fluid from mice expressing high levels of GM-CSF (29). Although cells with DC function and surface phenotype could be segregated from macrophages at the extremes of the distribution, there appeared to be a continuum of phenotypes rather than two discrete populations. For us the clarification came when, with Naik, the immediate precursor of the spleen cDC was isolated and shown to be distinct from monocytes and unable to produce macrophages (16). We termed these pre-DCs. This led to the view that there were two different routes to cells with DC antigen presenting function, one via monocytes and more often found under conditions of inflammation, the other derived from CDP/pro-DC precursors in bone marrow then via pre-DC to the types of DC found in steady state lymphoid tissue (17). The culture model finally developed for generation of the type of DCs found in steady state became bone marrow stimulated with Flt3 ligand, rather that with GM-CSF (30, 31). Thus, the developmental pathway leading to DC functions became a major criterion for segregating and naming DC subtypes.

It was then possible to segregate DCs derived from monocytes from the cDCs found in steady state spleen. However, it is evident from the account above that our previous nomenclature of the subsets of spleen cDCs based on CD8α expression was inadequate. Certain pDC subsets also expressed CD8α. Early DCs of “CD8^+^ cDC” lineage in spleen did not express CD8α. The migratory version of the same lineage, the CD103^+^ DCs, did not express it. And finally, CD8 was not expressed by human DCs. A major advance was the demonstration in several laboratories of an equivalent of the mouse “CD8^+^ cDC” lineage within human DCs, and the finding that the chemokine receptor XCR1 and the C-type lectin-like molecule Clec9A, rather than CD8, served as common DC surface markers crossing this species barrier [commentary in Ref. (32)]. The proposed designation of this DC subtype as DC1 overcomes the previous nomenclature problems (33).

In contrast to these advances in understanding the DC1 subset, the CD8^−^ CD11b^high^ SIRPα^high^ cDCs (designated as DC2) have been less studied and still present nomenclature issues. We had already separated spleen CD8^−^ DCs into two subsets based on CD4 expression (5), but the significance of this remains obscure. A more meaningful separation can now be made based on surface expression of Clec12A (DCAL2, MICL) versus DCIR2 or ESAM (6, 34, 35). An example of such segregation is shown in Figure 1. Importantly, these DC subsets differ in both developmental requirements and immunological characteristics; formation of DCIR2^+^ ESAM^high^ Clec12A^−^ DCs requires Notch2 signaling and this subset selectively responds to flagellin and induces Th2 responses. Will these differences demand a further division into DC2 and DC3 subtypes? Or will one of these, particularly the Clec12A^+^ subset, prove to be part of the monocyte-derived group? These questions require further work.

It is notable that ontogeny has led to a better understanding and provided one logical basis for DC classification (33). Will ontogeny be the best guide for DC nomenclature in future? We can foresee one area where it may cause confusion. A proportion of mouse pDCs and the CD8α-expressing subset of cDCs in the mouse thymus have a potential route of development from lymphoid rather than myeloid precursors (36, 37). These DC types have D–J rearrangements in their Ig heavy chain genes, a characteristic of lymphoid-origin cells (38). The extent to which a lymphoid route contributes to their development in steady state is still unclear, but the potential is there. Yet, the thymic CD8^+^ DCs are similar to the splenic CD8^+^ DCs of myeloid origin, and pDCs developing from myeloid or lymphoid precursors have similar surface phenotype and immunological functions. Should they have separate names according to their developmental origin, or should this “convergent” development lead to cells with the same name? There may yet be fine differences in function that eventually will be important to specify, but at present they are called by the same name. One resolution of this paradox comes from the likelihood that, despite the differences in bone marrow precursor surface markers, a common molecular program for pDC or for CD8^+^ cDC formation has been initiated, with transcription factors that override any previous precursor orientation. Considering ontological origin in terms of these final molecular programs, rather than by the surface markers on the precursor cells, should overcome the paradox resulting from apparent convergent differentiation.

## Conflict of Interest Statement

The authors declare that the research was conducted in the absence of any commercial or financial relationships that could be construed as a potential conflict of interest.
